# 1905

**Published:** 1905-12-30

**Authors:** 


### The Hospital.

A Journal of

The Medical Sciences and Hospital Administration.

Vol. XXXIX.—No. 1,005. SATURDAY, DECEMBER 30, 1905.

### 1905.

Although the artificial divisions of time produce no break in the actual continuity of events, and although the passage from December to January is marked by no phenomena calculated to arrest ordinary attention, the practice of regarding the close of the year as an epoch has become far too well established to be lightly laid aside. We cannot but be conscious that it affords a convenient standpoint from which to reconsider our relations with our environment, to calculate our losses or our gains, to meditate upon the results of another year's experience, and to think out plans for the guidance of our future conduct, or for the better promotion of our future enterprises. The time is one for good resolutions, and for the hope that the close of the approaching year may see at least some of them fulfilled.

While such is the relation of the event to individuals, it obviously affords reasons for somewhat similar conduct in the case of the aggregates of men who constitute professions, and who are called upon to think collectively upon a variety of topics. Of these, so far as the profession of medicine is concerned, it can hardly be said that any of absorbing interest have been brought into special prominence during the year now coming to a close. It is proverbially true, however, that the real significance of passing events is not always discernible by contemporaries; and we are somewhat inclined to think that the year in which the conduct of preliminary scientific education for the profession was abandoned by some of the metropolitan medical schools, and committed to the care of the University of London, may not only be hereafter regarded as one worthy of being held in remembrance, but also as one in which the first steps were taken towards the accomplishment of a real and noteworthy reform. The tendency of the change will clearly be in the direction of placing medical studies upon a new and more secure foundation than has ever hitherto been given to them, and of establishing the essential unity which underlies the occurrence of natural phenomena in all their forms. It may fairly be contended that the scope of medical education in the past has been too restricted; and that its aims in the future should be so enlarged as to bring under review the intimate connection which unites all the operations of Nature. Disease must no longer be regarded as an isolated fact, but must be viewed in its due connection with long chains of causation and of effect.

Another event of the passing year which in time to come will probably be looked upon as noteworthy is the progress which has been made towards the attainment of actual remedies or antidotes against some of the most destructive and the most widely operative of the causes of disease. The labours in this direction of Professor A. E. Wright, Dr. David Lawson, Dr. I. S. Stewart, Mr. J. G. Pardoe, and others, fully justify the hope that the tubercle bacillus will at no distant time be dealt with by methods directly destructive of its vitality; and that the diseases produced by it, which at present we can do little more than aid the natural forces to resist, will be prevented by meeting their causes in the very first stage of their operation. The hopes held out by Dr. Behring, at the Paris Conference, were hardly expressed in terms of sufficient decision to justify great reliance upon them, notwithstanding the high and well-deserved repute of their author; and it may perhaps almost be regretted that he did not practise greater reticence in their expression. But, whether they be fulfilled in his hands or not, the fact remains that the attainment of the ends which he proposes seems to be within the horizon of scientific outlook, and that the prospect thus opened points to the imperative necessity, for all labourers hoping to attain to it, of a scientific as well as of a clinical education. The path towards discovery is beset with errors, and the observer unassisted by really scientific training will be in constant danger of straying in pursuit of unverified hypotheses. The imagination of those who are in search of truth must be constantly controlled by the caution which only scientific training can impart; and the effects of the premature disclosure of Professor Koch's hopes from tuberculin may well serve as a lesson to restrain others from similar and perhaps even more disastrous errors.

Another event of 1905 has been the awakening of public and professional interest in the question of the physical condition of the nation as a whole, and the steps which have consequently been taken to discover the facts by actual observation and measurement. It is as true to-day as it was in the time of the Trojan war that physical force is the ultimately underlying condition of national predominance; and the state of large numbers of our soldiers in the South African war was certainly such as to justify the fear that the physical force of the nation has fallen greatly below the standard which at one time it maintained. The causes of such a declension, assuming it to be real, are much more easy to find than the remedy for them; because they clearly depend, in the main, upon the self-indulgent habits of ignorant people, accustomed, under our policy, to greater freedom of action than they can use either honestly or wisely. The freedom to be dirty, the freedom to be idle, the freedom to waste in beer and tobacco, or to lose in gambling, the money which ought to support a family in decency, the permitted ignorance of food values, of household economy, of child-rearing, of the causes of disease—these, in the long run, are the sources of any physical degeneration which may exist. They are causes not likely to be effectively remedied, as long as venal politicians depend for place upon the votes of numerical majorities living in insanitary conditions, and "educated," so to speak, in schools controlled for political ends.

